# Prevalence, Clinical Predictors, and Outcome of Hypocalcaemia in Severely-malnourished Under-five Children Admitted to an Urban Hospital in Bangladesh: A Case-Control Study

**Published:** 2014-06

**Authors:** Mohammod J. Chisti, Mohammed A. Salam, Hasan Ashraf, A.S.G. Faruque, Pradip K. Bardhan, Abu S.M.S.B. Shahid, K.M. Shahunja, Das Sumon K., Tahmeed Ahmed

**Affiliations:** ^1^Clinical Services, icddr,b, GPO Box 128, Dhaka 1000, Bangladesh; ^2^Centre for Nutrition & Food Security (CNFS), icddr,b, GPO Box 128, Dhaka 1000, Bangladesh; ^3^Research & Clinical Administration and Strategy, icddr,b, GPO Box 128, Dhaka 1000, Bangladesh

**Keywords:** Children, Hypocalcaemia, Severe malnutrition, Bangladesh

## Abstract

Hypocalcaemia is common in severely-malnourished children and is often associated with fatal outcome. There is very limited information on the clinical predicting factors of hypocalcaemia in hospitalized severely-malnourished under-five children. Our objective was to evaluate the prevalence, clinical predicting factors, and outcome of hypocalcaemia in such children. In this case-control study, all severely-malnourished under-five children (n=333) admitted to the Longer Stay Ward (LSW), High Dependency Unit (HDU), and Intensive Care Unit (ICU) of the Dhaka Hospital of icddr,b between April 2011 and April 2012, who also had their total serum calcium estimated, were enrolled. Those who presented with hypocalcaemia (serum calcium <2.12 mmol/L) constituted the cases (n=87), and those admitted without hypocalcaemia (n=246) constituted the control group in our analysis. The prevalence of hypocalcaemia among severely-malnourished under-five children was 26% (87/333). The fatality rate among cases was significantly higher than that in the controls (17% vs 5%; p<0.001). Using logistic regression analysis, after adjusting for potential confounders, such as vomiting, abdominal distension, and diastolic hypotension, we identified acute watery diarrhoea (AWD) (OR 2.19, 95% CI 1.08-4.43, p=0.030), convulsion on admission (OR 21.86, 95% CI 2.57-185.86, p=0.005), and lethargy (OR 2.70, 95% CI 1.633-5.46, p=0.006) as independent predictors of hypocalcaemia in severely-malnourished children. It is concluded, severely-malnourished children presenting with hypocalcaemia have an increased risk of death than those without hypocalcaemia. AWD, convulsion, and lethargy assessed on admission to hospital are the clinical predictors of hypocalcaemia in such children. Presence of these features in hospitalized children with severe acute malnutrition (SAM) should alert clinicians about the possibility of hypocalcaemia and may help undertake potential preventive measures, such as calcium supplementation, in addition to other aspects of management of such children, especially in the resource-poor settings.

## INTRODUCTION

Severe malnutrition is a burning child health problem in developing countries, including Bangladesh. It is associated with high morbidity and deaths (1). With the exception of sodium, severely-malnourished children have deficiency of total body electrolytes, such as potassium, magnesium, and calcium (2). The World Health Organization (WHO) recommends the routine use of potassium and magnesium supplementation in severely-malnourished children (3) but there is still no recommendation for calcium supplementation, although F-75 and F-100 in stabilization and nutritional rehabilitation phases respectively contain 320 mg calcium/litre. Hypocalcaemia is often associated with serious consequences, such as seizures and death (4,5), especially in children with severe malnutrition (6). Clinical manifestations of hypocalcaemia in severely-malnourished children are often subtle due to reduced muscle power (7) and overlap with the clinical signs of hypokalaemia and hypomagnesaemia (8,9). In addition, hypomagnesaemia is often associated with hypocalcaemia, leading to fatal seizures in children (8,10). Thus, the lack of supplementation of calcium in children with severe malnutrition may impede and/or delay recovery from the potential consequences of hypocalcaemia in such children. A better understanding of the predictors of hypocalcaemia in this population may improve the use of calcium, and simultaneously, may help reduce hypocalcaemia-related morbidity and mortality, especially in a resource-poor setting. However, there are no published data on the prevalence, clinical predictors, and outcome of hypocalcaemia in severely-malnourished children.

Like other hospitals in Bangladesh and in other developing countries, children often present to the Dhaka Hospital of icddr,b, with combinations of diarrhoea, malnutrition, and electrolyte disturbances, including hypocalcaemia. The objectives of our study were to identify the prevalence, clinical predictors, and outcomes of hypocalcaemia in inpaitent under-five children with severe malnutrition at the Dhaka Hospital of icddr,b.

## MATERIALS AND METHODS

### Study design

Children of either sex, aged 0-59 month(s), with severe malnutrition (who had their total serum calcium measured) admitted to the Longer Stay Ward (LSW), High Dependency Unit (HDU), and Intensive Care Unit (ICU) of the Dhaka Hospital of icddr,b between April 2011 and April 2012 were included in the study (n=333). In this case-control study, children with a biochemical evidence of hypocalcaemia (serum calcium <2.12 mmoL/L) constituted the cases, and those admitted without hypocalcaemia (serum calcium ≥2.12 mmoL/L) formed the control group. Children with severe wasting (weight-for-height z-score <-3 of the median of the WHO anthropometry) or severe undernutrition (weight-for-age z-score <-4 of the median of the WHO anthropometry), or nutritional oedema were considered severely-malnourished.

### Setting

Each year, the Dhaka Hospital of icddr,b, situated in the capital city of Bangladesh, provides care and treatment to around 140,000 patients of diarrhoea with or without associated complications, such as dyselectrolytaemia, including hypocalcaemia, and with or without other health problems, such as malnutrition. Diarrhoeal children with such complications or associated problems are admitted either to the LSW or ICU of the hospital. The vast majority of the patients are from poor socioeconomic backgrounds and live in urban or peri-urban Dhaka.

### Patient management

All the study children receive care and treatment which include supportive care, including intravenous fluids, antibiotics, oxygen therapy, frequent monitoring and assessment, and nutritional support (breastmilk if available, micronutrients, zinc, or culturally-acceptable food appropriate for age).

Antibiotics were administered to all study children as all of them had severe malnutrition. They received oral rehydration salts solution (ORS) by nasogastric tube or intravenous (IV) fluids, if they were severely dehydrated or had septic shock. Oral correction for dehydration was done by ORS, and intravenous correction was done by either cholera saline (sodium 133 mmol/L, potassium 13 mmol/L, chloride 99 mmol/L, and bicarbonate as sodium acetate 48 mmol/L) or normal saline (0.9% sodium chloride: sodium 154 mmol/L and chloride 154 mmol/L). Management of protein-energy malnutrition followed the icddr,b hospital's guidelines (3,11), and management of hypocalcaemia was done with the oral supplementation (or through nasogastric tube) of calcium (100 mg two to three times per day) for at least one week, which was often required to achieve normal serum calcium level.

### Measurements

Case report forms (CRF) were developed, pretested, and finalized for acquisition of data. Parameters that were analyzed included sociodemographic characteristics (age, gender, poor socioeconomic status, and stopping of breastfeeding during neonatal period), history and clinical signs [acute watery diarrhoea (AWD), vomiting, lethargy, dehydration (some/severe)] as defined by ‘Dhaka Methods’ that is almost similar to the WHO method and approved by WHO (12), convulsion at admission, respiration rate, diastolic blood pressure, abdominal distension, and hypoxaemia], severe sepsis, results of laboratory tests (total serum calcium, serum magnesium, serum sodium, potassium, chloride, and total carbon dioxide), and outcome.

Severe sepsis was defined as the presence of poor peripheral perfusion [absent peripheral pulses, and/or hypotension (blood pressure less than 50th centile of the WHO standard), with or without capillary refilling time >2 seconds] in absence of clinical dehydration or after correction of dehydration plus sepsis (13). Sepsis was defined as the presence of inflammation [abnormal WBC count (>12×10^9^/L, or <4×10^9^/L, or band and neutrophil ratio ≥0.1)] plus presence or presumed presence of infection with thermo-instability [hypo (≤35.0 ºC) or hyperthermia (≥38.5 ºC)], and tachycardia (13,14).

### Ethics

The study (Protocol number: PR-10067) was approved by the Research Review Committee (RRC) and the Ethical Review Committee (ERC) of icddr,b, and a written informed consent was obtained from parents or guardians before enrollment of children in the study. Children whose parents/caregivers did not provide consent for enrollment were not included in the study.

### Analysis

All data were entered into a personal computer (PC), using SPSS for Windows (version 17.0; SPSS Inc., Chicago, IL, USA) and Epi Info (version 6.0, USD, Stone Mountain, GA, USA). Differences in proportions were compared by the chi-square test. Differences in means were compared by Student's *t*-test for normally-distributed data or Mann-Whitney test for data that were not normally distributed. A probability of less than 0.05 was considered statistically significant. Strength of association was determined by calculating odds ratio (OR) and 95% confidence interval (CI). In analyzing predictors of hypocalcaemia in SAM children, likely variables were initially analyzed in a univariate model, and then the independent predictors were identified after controlling for the covariates in a logistic regression model.

## RESULTS

Out of 333 severely-malnourished children enrolled in the study, 87 (26%) and 246 (74%) were the cases and the controls respectively. The cases had higher probability of having a fatal outcome compared to the controls (17% vs 5%; p<0.001) (Table 1). Overall, 241/333 (72%) children in our study population had both severe wasting and severe underweight. The cases more often had vomiting, abdominal distension, lower diastolic blood pressure, hypomagnesaemia, severe sepsis, and bacteraemia compared to the controls (Table 1). The median age, gender distribution, distribution of poor socioeconomic status, stopping of breastfeeding during neonatal period, clinical dehydration, respiration rate, and hypoxaemia were comparable among the groups (Table 1). The cases frequently presented with more severe form of wasting than the controls (Table 1). Underweight and electrolytes were equally distributed among the groups (Table 1). In logistic regression, after controlling for potential confounding factors, such as vomiting, abdominal distension, and diastolic hypotension, the independent clinical predictors for hypocalcemia were AWD, convulsion on admission, and lethargy (Table 2).

## DISCUSSION

The study found significantly higher case-fatality rate among severely-malnourished children who had hypocalcaemia compared to those without hypocalcaemia. The hypocalcaemic severely-malnourished children had greater evidence of severe form of bacteraemia with systemic manifestation, such as severe sepsis, abdominal distension, and vomiting compared to their non-hypocalcaemic counterparts. This corroborate findings in recent publications that suggest co-morbidity of severe sepsis, abdominal distension, and vomiting. The potential consequences of bacteraemia in severely-malnourished children are often associated with high case-fatality rate (15-18), and this explains the observation of higher case-fatality rate among hypocalcaemic severely-malnourished children in our study.

AWD, convulsion on admission, and lethargy have been identified as the independent predictors of hypocalcaemia in SAM children. Our observation of low serum magnesium in severely-malnourished children with hypocalcaemia compared to those without hypocalcaemia often indicates significant intracellular depletion of magnesium (19). Severely-malnourished children often have deficiency of total body magnesium (2); however, loss of magnesium in stool during diarrhoea is likely to play a significant role in further depletion of intracellular magnesium (19,20). This, in fact, is one of the important factors in the pathogenesis of hypocalcaemia (21,22) and might result from either increased calcium influx into bone or skeletal resistance to parathyroid hormone (23). This might explain our frequent observation of association of AWD with hypocalcaemia in severely-malnourished children. However, in a few cases, severely-malnourished children may present with chronic severe hypocalcaemia and may remain asymptomatic (7).

**Table 1.** Clinical characteristics of under-five children with severe acute malnutrition (SAM) with hypocalcaemia (cases) and without hypocalcaemia (controls)

Characteristics	Cases (n=87)	Controls (n=246)	OR/ MD	95% CI	p value
Male gender	49 (56)	134 (55)	1.08	0.64,1.82	0.863
Age in months (median, IQR)	10.0 (5.0,18.0)	10.0 (4.7,15.3)	−	−	0.334
Poor	72 (83)	208 (85)	1.14	0.56,2.29	0.824
Stopped breastfeeding at neonatal period	32 (37)	64 (26)	1.65	0.95,2.88	0.077
Acute watery diarrhoea (AWD)	75 (86)	169 (69)	2.85	1.41,5.88	0.002
Vomiting	20 (23)	26 (11)	2.53	1.26,5.04	0.006
Lethargy	27 (31)	26 (11)	3.81	1.98,7.32	<0.001
Clinical dehydration (some/severe)	16 (18)	25 (10)	1.99	0.95,4.14	0.069
Convulsion at admission	11 (13)	3 (1)	11.72	2.93,54.48	<0.001
Respiration rate/minute (mean±SD)	46.9±10.8	48.0±11.9	−2.51	−5.38,+0.36	0.087
Diastolic blood pressure (mm of Hg) (mean±SD)	53.3±13.1	57.8±12.4	−4.53	−7.63,-1.44	0.004
Severe sepsis	10 (12)	9 (4)	3.39	1.22,9.48	0.013
Abdominal distension	18 (21)	25 (10)	2.31	1.13,4.70	0.019
Hypoxaemia (SpO_2_ <90%)	13 (15)	23 (9)	1.70	0.77,3.73	0.214
Hypomagnesaemia (serum magnesium <0.7 mmol/L)	7 (8)	4 (2)	5.29	1.35,22.16	0.009
Bacteraemia	14 (16)	18 (7)	2.45	1.09,5.49	0.028
Outcome (died)	15 (17)	11 (5)	4.39	1.81,10.79	<0.001
Nutritional severity and electrolyte imbalance on admission				
Weight-for-age z-score (undernutrition) (mean±SD)	−5.25±2.10	−4.88±1.33	−0.76	−0.85,-0.10	0.121
Weight-for-length/height z-score (wasting) (mean±SD)	−4.40±1.69	−3.64±1.41	−0.38	−1.13,-0.40	<0.001
Serum sodium (mmol/L) (mean±SD)	137.64±14.12	136.73±9.00	0.90	−2.30,4.11	0.577
Serum potassium (mmol/L) (mean±SD)	4.72±1.03	4.17±1.27	0.54	−1.67,2.75	0.626
Serum chloride (mmol/L) (mean±SD)	111.77±15.87	108.78±12.40	2.99	−0.72,6.70	0.113
Serum total carbon dioxide (mmol/L) (mean±SD)	17.97±11.19	18.27±6.02	−0.30	−2.79,2.20	0.815

Figures represent n (%), unless specified; CI=Confidence interval; IQR=Interquartile range; MD=Mean difference; OR=Odds ratio; SD=Standard deviation. SpO_2_=Transcutaneously-measured arterial blood oxygen concentration by pulse oximeter

**Table 2.** Results of logistic regression to explore the independent clinical predictors of hypocalcaemia in under-five children with severe acute malnutrition

Predictor	OR	95% CI	p value
Acute watery diarrhoea (AWD)	2.19	1.08-4.43	0.030
Convulsion	21.86	2.57-185.68	0.005
Lethargy	2.70	1.33-5.46	0.006
Vomiting	1.95	0.95-4.01	0.071
Abdominal distension	1.54	0.72-3.28	0.264
Diastolic blood pressure	0.99	0.97-1.01	0.323

Reductions in serum calcium enhance motor nerve excitability, which is provoked by hypomagnesaemia, leading to a number of features, including twitching of muscles and seizure (23). The observation of seizure in our study population is consistent with a number of previous studies (10,24-26). Reduced diastolic blood pressure (diastolic hypotension) in significantly higher proportion of hypocalcaemic severely-malnourished children in the absence of dehydration and also after correction of dehydration in already dehydrated children is one of the markers of severe sepsis in under-five children (14,16). This indicates that higher proportion of severely-malnourished children with hypocalcaemia had severe sepsis that almost always is featured by altered mental status, including lethargy (6,21). Moreover, frequent observation of abdominal distension and vomiting in hypocalcaemic severely-malnourished children is the cardinal feature of ileus in such children (6,21), which possibly contributes to lethargy. This explains the frequent observation of lethargy in hypocalcaemic severely-malnourished children, which is consistent with earlier reports (21,23).

No difference was observed in the distribution of age, gender, poor socioeconomic status, stopping of breastfeeding during neonatal period, clinical dehydration, respiration rate, hypoxaemia, serum sodium, potassium, chloride, and total carbon dioxide among the groups. These would tend to suggest that the two populations in our study were derived from a single larger population and that the predictors of hypocalcaemia, as noted in our study, were not perhaps due to bias in selecting the cases and controls.

Our observation showed that hypocalcaemic children more often presented with severe form of wasting and bacteraemia compared to those without hypocalcaemia. This may suggest that hypocalcaemia might be a result of the co-morbidities of the severity of wasting (2) and bacteraemia (6,27). Although most of the study population with severe malnutrition also had severe underweight, suggesting a chronic form of malnutrition, it failed to show any significant association with the development of hypocalcaemia.

### Strengths and limitations

The main limitation of our study is the lack of measurement of ionized calcium which is a more accurate reflection of the physiologic calcium state (28). However, low serum albumin (not measured in the present study) as found in most of the severely-malnourished cases may show inappropriately higher level of ionized calcium (28). However, our main aim was to evaluate the simple clinical predictors of hypocalcaemia in children with severe malnutrition, and we feel that despite the limitations, the results still have the clinical relevance in resource-poor settings where there is lack of opportunity to assess total/ionized calcium.

### Conclusions

Based on the findings of our study, we may conclude that hypocalcaemic children with severe malnutrition are at higher risk of deaths compared to those without hypocalcaemia. AWD, convulsions, and lethargy on admission may be used as predictors of hypocalcaemia in severely-malnourished children and may help in decision-making for calcium supplementation, along with magnesium supplementation and other management following WHO guideline. This may be particularly useful in resource-poor settings where routine estimation of serum calcium and magnesium is not possible.

## ACKNOWLEDGEMENTS

This research was funded by icddr,b and its donors which provide unrestricted support to icddr,b for its operations and research. Current donors providing unrestricted support include: Australian Agency for International Development (AusAID), Government of the People's Republic of Bangladesh, Canadian International Development Agency (CIDA), Swedish International Development Cooperation Agency (Sida), and the Department for International Development (DFID), UK. We gratefully acknowledge these donors for their support and commitment to icddr,b's research efforts. We would like to express our sincere thanks to all physicians, clinical fellows, nurses, members of feeding team, and cleaners of the hospital for their invaluable support and contribution during patient enrollment and data collection.
